# Prevalence and Predictors of Postpartum Depression: Northwest Ethiopia

**DOI:** 10.1155/2020/9565678

**Published:** 2020-01-21

**Authors:** Mengstu Melkamu Asaye, Haymanot Alem Muche, Eyerusalem Desta Zelalem

**Affiliations:** ^1^MSc in Clinical Midwifery, School of Midwifery, College of Medicine and Health Science, University of Gondar, P.O. Box 196, Ethiopia; ^2^MSc in Clinical Midwifery, University of Gondar Comprehensive Specialized Hospital, P.O. Box 196, Ethiopia

## Abstract

**Background:**

Postpartum depression is an umbrella, which encompasses several mood disorders that follow childbirth within 6 weeks. Screening for postpartum depression would improve the ability to recognize these disorders and enhance care that ensures improved clinical outcomes. Early identification of postpartum depression is important in order to plan for implementation strategies that allow for timely treatment and support of women with postpartum depression.

**Objective:**

To determine the prevalence and associated factors of postpartum depression among women who gave birth in the last six weeks in Gondar town, Northwest Ethiopia, 2018.

**Methods:**

A community based cross-sectional study was conducted among 526 women who gave birth in the last 6 weeks from July 1 to 30, 2018 in Gondar town. Cluster sampling technique was used. Data were collected by semi-structured and pretested questionnaire and entered into epi-Info version 7.0 and then analyzed by SPSS version 20.0. Both bivariate and multivariable logistic regression model were fitted. Adjusted odds ratio with 95% confidence interval has been computed and variables with *p*-value <0.05 were considered statistically significant.

**Results:**

The prevalence of postpartum depression among 526 postnatal women was 25% (95% CI: 21, 28). Abortion history (AOR = 1.79, 95% CI: 1.07, 2.97), birth weight <2.5 kg (AOR = 3.12, 95% CI: 1.78, 5.48), gestational age below 36 weeks (AOR = 2.18, 95% CI: 1.22, 3.88) unplanned pregnancy (AOR = 2.02, 95% CI: 1.24, 3.31), relatives' mental illness (AOR = 1.20: 1.09–3.05), had no antenatal visit (AOR = 4.05, 95% CI: 1.81, 9.05), had no postnatal visit (AOR = 1.82, 95% CI: 1.11, 3.00) were factors significantly associated with postpartum depression. *Conclusion and Recommendations.* The prevalence of PPD was found to be higher. Variables like abortion history, low birth weight, gestational age below 36 weeks, unplanned pregnancy, relatives' mental illness, had no antenatal visit, and had no postnatal visit were predisposing factors to postpartum depression. Preventive measures to avoid low birth weight and pregnancy complications are also identified as proactive ways to reduce postpartum depression. Early identification and treatment of depression during ANC and postpartum care can mitigate the impact of PPD on the mother-baby dyad. Emphasis must be given women to have ANC and PNC follow up.

## 1. Introduction

Postpartum depression is a very common problem, which occurs in women of childbearing age within 6 weeks of childbirth, but is often unrecognized or undiagnosed and a significant public health burden [1]. Risk factors for postpartum depression include social stressors, such as poverty, intimate partner violence, history of pregnancy loss, unintended pregnancy, and these variables have adverse effects on maternal health [2]. An important distinction that makes postpartum depression unique from other depression disorders is that it is marked by prominent anxiety components [3]. The problem of postnatal depression not only has immediate adverse effects on the mother, her newborn, child, and the family but can also lead to long-term morbidity of chronic or recurring depression [4].

Current models of postpartum care in developed countries originated in the beginning of the 20^th^ century in response to the high maternal and neonatal mortality rates of the time [5]. Untreated postpartum depression can have adverse long-term effects. In addition to the maternal risk from chronic depression, offspring may exhibit emotional, behavioral, cognitive, and interpersonal impacts associated with their mother's depression [6].

Four million births occur in the world annually; approximately forty percent of new mothers are affected with different types of postpartum mood disorders including depression symptoms before and during pregnancy [7].

The relevance of this research on PPD establishes not only the regional prevalence of the disorder but identifies proactive approaches to both identify the disorder and modify the complications of PPD. The endpoint is to mediate the impact of PPD on the mother and child dyad, the family, and the community.

The prevalence of postpartum depression varies from 1.9% to 82.1% in developing countries [8]. Global studies show differing prevalence rates, with researchers in Canada reporting a prevalence of 40% [9], and in Jamaican 56% and 34% depression prevalence during prepartum and postpartum periods, respectively [9]. Studies show that the prevalence of postpartum depression is 9.2% in Sudan [10]. In addition, in a study carried out in eastern Tigray zone, Ethiopia, the prevalence of major depression at six weeks postpartum was 19% [11].

The effects of postnatal depression on the mother, her marital relationship, and her children make it an important condition to diagnose, treat, and prevent. The provision of care will vary depending on the socio-demographic and obstetric factors. Globally, PPD prevalence and associated risk factors are important to identify, especially in low and middle income countries.

The aim of this research is to translate this information to health care workers and to women in order to improve care related to PPD. The identification of the magnitude and risk factors that determine postpartum depression will help health care providers and other concerned bodies to suggest the diagnosis and interventions to be designed for the neglected postpartum depression. Postpartum depression is a neglected problem. It causes a lot to maternal ill health, jeopardizes the newborn health, and quite often destablizes the family. The study will also provide greater inputs to the government and program managers for designing of programs, proper implementation, and evaluation of their contribution for the achievement of the new adopted agenda as Sustainable Development Goals (SDGs) in relation with postpartum depression.

## 2. Method

### 2.1. Study Design & Period

A community based cross-sectional study design was conducted from July 1 to 30, 2018.

### 2.2. Study Area

The study was conducted in Gondar town which is located 750 km from Northwest of Addis Ababa, the capital city of Ethiopia. According to the 2017 population projection, the total population size of Gondar town was estimated to be 338,646. From the total population of Gondar city 23.58% were women in the reproductive age group. The city has one comprehensive specialized hospital, 8 health centers, and three private maternity specialty clinics that give maternity services and one private primary hospital.

### 2.3. Source Population

All women who gave birth in Gondar town.

### 2.4. Study Population

All women who gave birth in the last 6 weeks prior to the study period and reside more than 6 months in the selected Kebele of Gondar town.

### 2.5. Inclusion Criteria

All mothers who gave birth 6 weeks prior to the data collection period at the selected kebeles of Gondar city.

### 2.6. Sample Size Estimation

The sample size was determined by considering the assumptions for single proportion formula.

(1) $$\begin{array}{c} n=\frac{{\left(za/2\right)}^{2}\cdot p\left(1-p\right)}{w^{2}}, \end{array}$$

*n* = initial sample size,

*a* = level of significance,

*p* = prevalence of ante partum depression (19%) [12],

*w* = marginal error of 5%,

(2) $$\begin{array}{c} n=\frac{{\left(1.96\right)}^{2}\cdot 0.19\left(1-0.19\right)}{{\left(0.05\right)}^{2}}=236. \end{array}$$

By assuming 2 design effect and 10% of nonresponse rate.

Sample size was =519 but I have got 7 additional participants in the cluster. The final sample size was 526.

### 2.7. Sampling Procedure

**Figure 1:**
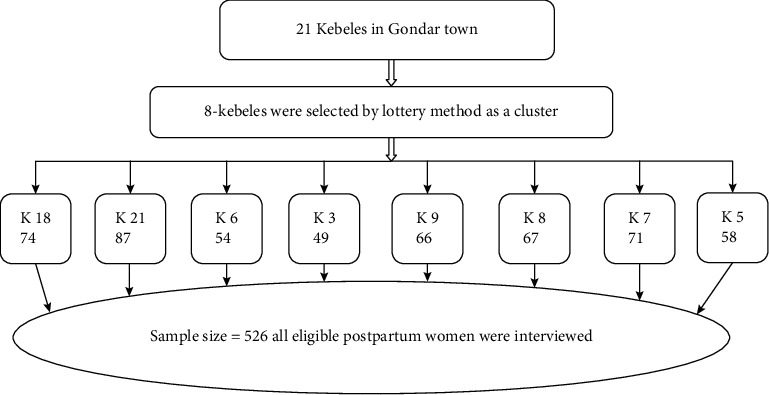
Schematic presentation of sampling procedure to assess prevalence and associated factors of postpartum depression among women who gave birth in the last six weeks at Gondar town, Northwest Ethiopia, 2018 (*n* = 526).

Gondar town has a total of 21 kebeles. The reference population was homogeneous, we use those 21 kebeles as a cluster; from these 8 kebeles were selected randomly using the lottery method. Then from each selected clusters, we took all women who gave birth in the last six weeks. Then the data collector used map and location of the urban health extension workers to get selected mothers as shown in the following (Figure 1).

### 2.8. Variables of the Study

#### 2.8.1. Dependent variable

Postpartum depression

#### 2.8.2. Independent variable

Socio-demographic variables: age, ethnic group, marital status, educational level, residence.

Obstetric variables: parity, premature labor, unplanned pregnancy, losing or hospitalizing a baby, mode of delivery, pregnancy complication or illness, stressful life event during pregnancy, experienced death of baby, and undesired fetal sex. Previous psychiatric history: history of depression and family history of psychiatric problems, and social support.Social support: poor husband support, domestic violence, child birth without the presence of any relatives, unsatisfactory relationship with mother-in-law, unsatisfactory relationship with husband.

### 2.9. Operational Definitions

#### 2.9.1. Postpartum Depression (PPD)

Postpartum depression is a psychiatric disorder that occurs in the women following delivery up to six weeks if ≥13 according to (EPDS). In the scale, the question numbers 1, 2, & 4 are scored 0, 1, 2, & 3 with first choice scored as 0, and the last choice scored 3, and question numbers 3, 5–10 are reversely scored with the first choice as 3 and the last choice scored as 0. After adding up all the scores, women who scored greater than or equal to 13 were concluded to have postpartum depression.

#### 2.9.2. History of Depression

Women who had mental illness that can interfere with a person's life. Sever feelings of sadness, hopelessness, and loss of interest in activities.

#### 2.9.3. Happy Spouse

Happy relationship/marriage with husband.

#### 2.9.4. Data Collection Tools and Procedures

Data were collected through pretested semi-structured face to face interview. The semi-structured questionnaires were prepared in local language, Amharic, to make it simple and understandable. One diploma midwifery student in each kebele for data collection and two BSc midwifery supervisors, total of 8 data collectors and 2 supervisors were recruited.

#### 2.9.5. Data Quality Control

The questionnaire was prepared in English, and then translated to Amharic (local language) and back to English to maintain consistency of the tool. Training was provided for data collectors and supervisors for one day about the purpose of the study and techniques of data collection. The trained data collectors were supervised during data collection and each questionnaire was checked for completeness in a daily basis. Data entry was conducted and it was cross checked. The questionnaire was pretested to check the response, language clarity, and appropriateness of the questionnaire, while the pretest was done outside study area at Maksegnit city with 5% of sample size. At the end of the pretest depending on its outcome, the correction measures were under taken.

#### 2.9.6. Data Processing and Analysis

Data cleaning was performed to check for accuracy, completeness, consistencies, and missed values and variables. After the data had been checked for completeness and accuracy, it was coded manually and then entered to Epi Info version 7.0 and exported to Statistical Package of Social Science (SPSS) version 20 for analysis. Descriptive statistics was performed on numerical value, standard deviation, frequencies, proportion to describe study population in relation to dependent and independent variables. Binary logistic regression was used to identify statistically significant independent variables and independent variables having *p*-value less than 0.2 will go to multivariable logistic regression for further analysis. Adjusted odds ratio with 95% confidence interval was used to determine the degree and direction of association between covariates and the outcome variable. To adjust for confounding variables, a multivariable logistic regression was done, model fitness was checked using Hosmer–Lemeshow goodness of fit test, and a *p*-value <0.05 with 95% confidence interval for odds ratio (OR) was used to determine significance.

#### 2.9.7. Ethical Clearance

Ethical clearance was obtained from the Institutional Review Board (IRB) of University of Gondar on behalf of the Ethics Review Committee of the department of midwifery. A letter of cooperation was obtained from Gondar town health office. The reasons why the research was to be done was explained to the study subjects; informed consent was obtained from each study subject after explanation of the purpose of the study, and involvement (to be participant) was after their complete consent. Any mother who was not willing to participate in the study was not forced to participate, no personal identifications were included in the data sheet, and all data taken from the participants were kept strictly confidential and used only for the study purpose.

## 3. Result

### 3.1. Socio-Demographic Characteristics of Participants

A total of 526 women were enrolled and the response rate was 100%. Mean age of the study participants was 28.7 years (standard deviation = 5.2) and 65.6% of them were orthodox Christians. More than three-fourths of the participants were married (80%). Nearly two-thirds of the respondents were (69.2%) up to intermediate in education and 42% of them were housewife by occupation. On the other hand, 68.9% of their husbands had working job (Table 1).

### 3.2. Obstetrics Related Characteristics of Respondents

More than one half (62%) of respondents had given birth below three times, while 65.2% of them had two or more living children. Fifty six (38.6%) of respondents had faced severe headache complication in the indexed pregnancy. Nearly half of the study subjects (58.56%) gave birth spontaneously vaginally. Around one half of the participants (50.8%) had four or more antenatal care whereas 53.5% of women had one postnatal care visit. Moreover, 15.2% of the indexed neonates were admitted to neonatal intensive care unit (Table 2).

### 3.3. Depression Related Characteristics of Respondents

Among the respondents, 46 (8.7%) of them had relatives with a mental illness history. Out of these, 60% of the women reported that her mother had mental illness problem. Sixty-three (12%) of the respondents had history of depression. Nearly three quarters of the candidate's husband (76%) were happy with their current marital status (Table 3).

### 3.4. Social-Support Related Characteristic of Respondents

Among the respondents, 19.2% of them were abused by their husbands. The common type (71.3%) of the abuse was verbal in kind. Most of the women relatives (88.8%) were present in health facilities in the indexed child birth. On the other hand, (55.3%) the respondents had happy relationship with their husband's family (Table 4).

### 3.5. Prevalence of Postpartum Depression

**Figure 2:**
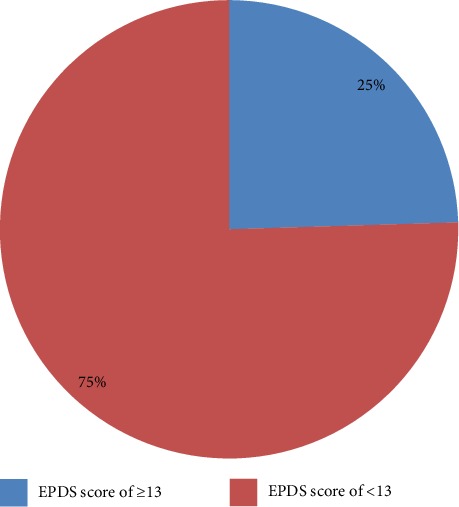
Prevalence of postnatal depression among women who gave birth in the last 6 weeks in Gondar town, Northwest Ethiopia, 2018 (*n* = 526).

From the entire respondents the prevalence of postpartum depression was found to be 25% (95% CI: 21–28) as shown in Figure 2.

### 3.6. Factors Associated with Postpartum Depression

The bivariate logistic regression analysis revealed that husband's occupation, income, live children, women who had abortion history, weight of newborn infant, gestational age at birth, planned pregnancy, complication during last pregnancy, ANC visit, PNC visit, history of relatives' mental illness, previous history of depression, happy with spouse, and relatives present at health facilities in the indexed delivery had association of postpartum depression at *p*-value less than 0.2.

Concerning multivariable logistic regression analysis, abortion history, birth weight, gestational age, planned pregnancy, ANC visit, history of relatives' mental illness, and women who had PNC visit were found to be significant associated factors of postpartum depression at *P*-Value <0.05.

According to this study, the odds of developing postpartum depression among women who ever had abortion were nearly two times than those who had no abortion history (AOR = 1.79, 95% CI: 1.07, 2.97).

In the current study, women who had low birth weight (<2.5 kg) new born were three times more likely to develop postpartum depression as compared to their counter parts (AOR = 3.12, 95% CI: 1.78, 5.48).

In this statistical analysis, respondents who gave birth below 36 weeks of gestational age were two times more likely to develop postpartum depression as compared to women who delivered at gestational age of 36 or more weeks (AOR = 2.18, 95% CI: 1.22, 3.88).

It was noted that unplanned pregnancy has been associated with the development of postpartum depression. The odds of developing postpartum depression among respondents who had unplanned pregnancies were two times than those women who had planned pregnancies (AOR = 2.02, 95% CI: 1.24, 3.31).

Participants who did not have antenatal visits were exposed to postpartum depression development. The study revealed that respondents who did not have antenatal visits were four times more likely to be depressed compared to those who had antenatal follow-up (AOR = 4.05, 95% CI: 1.81, 9.05). There is also a strong relationship between postnatal visit and postnatal depression. In this study, the odds of developing postpartum depression among respondents who did not have postnatal visits were nearly two times more likely to be depressed than those who had postnatal visits (AOR = 1.82, 95% CI: 1.11, 3.00). The report also declared that the odds of developing postpartum depression among participants whose relatives had mental illness history were 1.2 times more likely to be depressed than those whose relatives did not have mental illness history (AOR = 1.20, 95% CI: 1.09, 3.05) (Table 5).

## 4. Discussion 

The aim of this study was to assess the prevalence and associated factors of postpartum depression among women who gave birth in Gondar town, Ethiopia.

The study evidenced that the prevalence of postpartum depression was found to be 25%. This finding is in accordance with studies conducted in Iran—23.2% [12] and 28.1% in the Trabzon province [13]. However, the reports of the current study were found to be higher compared to studies done in Canada 8.69% [14], New Delhi 12.75% [15], Kampala-Ugandan urban primary health care 6.1% [16], Gujarati 12.5% [1], Harare Ethiopia 13% [17], and 19% prevalence in the eastern zone of Tigray [11]. The possible explanation might be the differences in study setting and type of design utilized. The other difference might be 68.2% of the respondents in this study were secondary and above by education. Higher educational levels for mothers may contribute to reporting postpartum depression, as they are aware of their depression and are empowered to report the symptoms. This also attributes that women with good educational level may have intellectual skills and better copying strategies.

On the other hand, the estimate of postpartum depression in the current study was lower than the study conducted in Victoria South Australia 38.3% [18], Nepal University Hospital 29% [19], Rawalpindi General Hospital 33.1% [20], in Cape-Town 45.1% [21], in Turkey 32.1% [12], and 48.6% in Karachi, Pakistan [22]. The higher prevalence may be related to the site of delivery, as many studies listed were conducted in university hospitals and regional referral centers. The findings may also be impacted by those patients who have access to care and those who seek care for depression in the postpartum period. The other explanation might be due to socio-cultural differences of respondents. It is vital to consider the role of culture and the impact patient's beliefs and the cultural support for receiving help for postpartum depression.

Women who ever had abortion, birth weight below 2.5 kg, gestational age below 36 weeks, planned pregnancy, antenatal visit, relatives who had mental illness, and postnatal visit were factors significantly associated with development of postpartum depression.

According to this study, the odds of developing postpartum depression among respondents who ever had abortion were nearly two times more likely to be depressed compared to those who had no abortion history. This report was supported by studies conducted at Gujarati postpartum women [1] and rural southern Ethiopia [2]. This might be because of the fact that women who had abortion will pose different psychosocial problems and they might regret for fear of complication development during their life of pregnancy.

The study revealed that the odds of developing postpartum depression among respondents whose new born child with low birth weight were three times more likely to be depressed as compared those who had high birth weight (≥2.5 kg) of new born. This report was in accordance with studies conducted in Canada [23]. This could be that depressed or anxious women may have exaggerated pessimistic concerns about the health of their newborn compared with those women who are psychologically well. Lastly, it might be due to the reason that low birth weight is likely to cause women to feel depressed for the newborn and to feel that she has failed to fulfill the baby's needs during her life span of pregnancy.

In this statistical analysis, respondents who gave birth below 36 weeks of gestational age were two times more likely to be depressed compared to those who gave birth at gestational age of 36 or more weeks. This finding is supported by a study conducted in Canada [23], Istanbul, Turkey [12], Arabic women [24], and Harare, Ethiopia [11]. The study might be due to the uncertainty about the survival of the newborn and doubts about one's capacity to cope with the care of an abnormal or ill newborn child.

It was noted that unplanned pregnancy has been associated with the development of postpartum depression. The odds of developing postpartum depression among respondents who had unplanned pregnancies were two times more likely to be depressed compared those whose pregnancies were planned. The result was supported with studies conducted in Uganda, and Nigeria [11, 25], Istanbul, Turkey [26], Kuwait [1], and Harare, Ethiopia [11]. The possible explanation might be because in African cultures child bearing was so widely desirable that issues of whether a baby was wanted never arose, even though individual women might not be psychologically or economically prepared for pregnancy. An unplanned baby's birth is likely to affect the whole life and career plans of a woman. This unplanned life course may predispose women to develop depression in her postpartum period.

Participants who did not have antenatal visits were exposed for development of postpartum depression. The study revealed that respondents who did not have antenatal visits were four times more likely to be depressed compared to those who had antenatal follow-up. This report was in agreement with studies conducted at north Carolina, Colorado [27], Khartoum, Sudan [28] and Gondar University Hospital, Ethiopia [29]. The study results may be affected by the care given during the antenatal care visits, where counseling and anticipatory guidance is given by care providers to expectant mothers. The care may build maternal self-esteem and resiliency, along with knowledge about normal and problem complications to discuss at care visits. Mothers who value ANC care may also have different views on their right to mental and physical wellness, including access to care.

There is also a strong relationship between postnatal visit and postnatal depression. In this study, the odds of developing postpartum depression among respondents who did not have postnatal visits were nearly two times more likely to be depressed compared to those who had postnatal visits. Although assumed that accessing care would lead to increased diagnosis of postpartum depression, the postpartum visits may in fact provide guidance, reassurance, and appropriate referrals that are unavailable to those who do not seek care. Health care professionals have an ability to both educate and empower mothers as they care for their babies, their families, and themselves.

The report also declared that the odds of developing postpartum depression among participants whose relatives had mental illness history were 1.2 times more likely to be depressed compared to those whose relatives did not have mental illness history. This finding was in accordance with studies conducted in Istanbul, Turkey [26], Bahraini [1], and Arba Minch, Ethiopia. The links between genetic pre-disposition to mood disorders, considering both nature and nurture are important to address. PPD may be seen as a “normal” condition for those who are acquainted with relatives with mood disorders, especially during the childbearing years. Family history of mental illness can be easily elicited in the ANC first visit history. This information targets women who may have additional PPD risks and need special attention during the postnatal period.

## 5. Conclusion and Recommendation

The prevalence of postpartum depression was found to be higher compared to the national target value. Factors that are associated with increased PPD include women who have had abortion history, low birth weight newborn, gave birth prematurely, had unplanned pregnancy, had no ANC follow up, and had no PNC follow up. Women at increased risks also had relatives with mental health histories, and those with previous history for depression were more likely to have postpartum depression after their birth.

Early identification and treatment of depression during postpartum care can mitigate the impact of PPD on the mother-baby dyad. Emphasis must be given women to have ANC and PNC follow-up.

## Figures and Tables

**Table 1:** Socio-demographic and economic related characteristics of women in Gondar town who gave birth in the last 6 weeks, Northwest Ethiopia, 2018, (*n* = 526).

Variables	Frequency	Percent (%)
*Ethnicity*		
Amhara	394	74.9
Others∗	132	25.1
*Religion*		
Orthodox	345	65.6
Others∗∗	181	34.4
*Current maternal age (in years)*		
Below 30	299	56.8
30 and above	227	43.2
*Marital status*		
In marital relation	421	80.04
Not in marital relation	105	19.96
*Maternal education*		
Non educated	14	2.7%
Up to intermediate	364	69.2
College and above	148	28.1
*Husband education ( n* = 421* )*		
Noneducated	10	2.4
Inter-mediate	234	55.6
College and above	177	34
*Maternal occupation*		
House wife	221	42
Student	46	8.8
Employed	232	44.1
Daily labor	27	5.1
*Husband occupation ( n* = 421* )*		
Employed	290	68.9
Merchant	101	24
Daily labor	30	7.1
*Family income (ETB∗*		
<750 ETB	24	4.56%
750–1200 ETB	53	10.08%
>1200 ETB	449	85.36%

Other∗ = Kemanit, Tigre, Gurage, Agew; other∗∗ = Muslim, catholic and protestant; ETB∗ = Ethiopian birr.

**Table 2:** Obstetrics related factors of women in Gondar town who gave birth in the last 6 weeks, Northwest Ethiopia, 2018, (*n* = 526).

Variables	Frequency	Percent (%)
*Parity*		
Below 3	326	62
3 and above	200	38
*Live births*		
Below 2	183	34.8
2 and above	343	65.2
*Ever had abortion*		
Yes	117	22.2
No	409	77.8
*Total abortion ( n* = 117* )*		
Below two	110	94
Two and above	7	6
*Mode of delivery*		
Spontaneous vaginal	308	58.5
Cesarean section	186	35.4
Instrumental	32	6.1
*Birth weight*		
Below 2.5 kg	95	18
2.5 kg and above	431	82
*Gestational age in weeks*		
Below 36 weeks	86	16.3
36 and above weeks	440	83.7
*Planned pregnancy*		
Yes	382	72.6
No	144	27.4
*Complication in the indexed pregnancy*		
Yes	145	27.6
No	381	72.4
*Type of complication in the indexed pregnancy( n* = 145* )*		
Vaginal bleeding	50	34.5
Severe headache	56	38.6
Other∗	39	26.9
*ANC visit*		
Yes	484	92
No	42	8
*Number of ANC visit ( n* = 484* )*		
Below 4	238	49.2
4 and above	246	50.8
*Desired sex of the baby*		
Yes	203	38.6
No	323	61.4
*PNC visit*		
Yes	368	70
No	158	30
*Number of PNC visit ( n* = 368* )*		
Below 2	197	53.5
2 and above	171	46.5
*Newborn admitted*		
Yes	80	15.2
No	446	84.8

Other∗ = fever, swelling, blurring of vision and fetal movement.

**Table 3:** Depression related characteristics of women who gave birth in the last 6 weeks at Gondar town, Northwest Ethiopia, 2018, (*n* = 526).

Variables	Frequency	Percent (%)
*Relatives' mental illness history*				
Yes	46	8.7
No	480	91.3
*Who had mental illness history ( n* = 46* )*				
Mother	28	60
Other∗	18	40
*History of depression*				
Yes	63	12
No	463	88
*Happy with marriage*				
Yes	399	75.9
No	127	24.1

Other∗ = brother and sis.

**Table 4:** Social-support characteristics of respondents who gave birth in the last 6 weeks in Gondar town, Northwest Ethiopia, 2018, (*n* = 526).

Variables	Frequency	Percent (%)
*Abused by your husband*				
Yes	101	19.2
No	425	80.8
*Type of abuse ( n* = 101* )*				
Verbal	72	71.3
Physical	24	23.8
Other∗	5	4.9
*Presence of relatives at health institutions during delivery*				
Yes	467	88.8
No	59	11.2
*Happy relationship with your husband's families*				
Yes	291	55.3
No	235	44.7

Other**∗**  = verbal and physical.

**Table 5:** Associated factors of postpartum depression among women who gave birth in the last 6 weeks in Gondar town (*n* = 526), 2018.

Variable	PPD		COR [95% CI]	AOR [95% CI]
	Yes (%)	No (%)				
*Live birth*				
Below 2	52 (28.4)	131 (71.6)	1	1
2 and above	77 (22.4)	266 (77.6)	1.37 (0.48,1.10)	0.99 (0.55, 1.79)
*Ever had abortion*				
Yes	41 (35)	76 (65)	1.97 (1.26, 3.08)	1.79 (1.07, 2.97)∗
No	88 (21.5)	321 (78.5)	1	1
*Birth weight*				
Below 2.5 kg	45 (47.4)	50 (52.6)	3.72 (2.33, 5.94)	3.12 (1.78, 5.48)∗∗∗
2.5 kg and above	84 (19.5)	347 (80.5)	1	1
*Gestational age in weeks*				
Below 36	41 (47.7)	45 (52.3)	3.64 (2.25, 5.91)	2.18 (1.22, 3.88)∗
36 and above	88 (20)	352 (80)	1	1
*Planned pregnancy*				
Yes	72 (18.8)	310 (81.2)	1	1
No	57 (39.5)	87 (60.5)	2.82 (1.85, 4.30)	2.02 (1.24, 3.31)∗∗
*Complication in the indexed pregnancy*				
Yes	46 (31.7)	99 (68.3)	1.67 (1.09, 2.55)	1.19 (0.71, 1.97)
No	83 (21.8)	298 (78.2)	1	1
*ANC visit*				
Yes	100 (20.7)	384 (79.3)	1	1
No	29 (69)	13 (31)	8.6 (4.30, 17.08)	4.05 (1.81, 9.05)∗∗
*PNC visit*				
Yes	67 (18.2)	301 (81.8)	1	1
No	62 (43.1)	96 (56.9)	2.90 (1.92, 4.39)	1.82 (1.11, 3.00)∗
*Relatives' mental illness history*				
Yes	15 (32.6)	31 (67.4)	1.55 (0.81, 2.98)	1.20 (1.09, 3.05)
No	114 (23.8)	366 (76.2)	1	1
*History of depression*				
Yes	25 (36.8)	38 (63.2)	2.27 (1.31, 3.94)	1.35 (0.68, 2.67)
No	104 (22.5)	359 (77.5)	1	1
*Presence of relatives at institution during delivery*				
Yes	107 (22.9)	360 (77.1)	1	1
No	22 (37.3)	37 (62.7)	2.00 (1.13, 3.54)	0.97 (0.46, 1.85)

*Note.* ∗∗∗ = *P* value <0.001, ∗∗ = *P* value <0.01, ∗ = *P* value <0.05.

## Data Availability

The data used to support the findings of this study are available from the corresponding author upon request.

## Abbreviations

CI: Confidence interval
EPDS: Edinburgh postnatal depression scale
PPD: Postpartum depression
PND: Post natal depression
SPSS: Statistical package for social scientists
SSA: Sub saharan Africa
WHO: World health organization
ANC: Ante natal care
PNC: Post natal care.
