# Corrigendum to “Prevalence and Predictors of Postpartum Depression: Northwest Ethiopia”

**DOI:** 10.1155/2020/9084894

**Published:** 2020-09-10

**Authors:** Eyerusalem Desta Zelalem, Mengstu Melkamu Asaye, Haymanot Alem Muche

**Affiliations:** ^1^MSc in Clinical Midwifery, University of Gondar Comprehensive Specialized Hospital, P.O. Box 196, Ethiopia; ^2^MSc in Clinical Midwifery, School of Midwifery, College of Medicine and Health Science, University of Gondar, P.O. Box 196, Ethiopia

In the article titled “Prevalence and Predictors of Postpartum Depression: Northwest Ethiopia” [1], the order of the authors in the author list was incorrect. The correct order is shown below, and is updated in the author list above:

Eyerusalem Desta Zelalem, Mengstu Melkamu Asaye, Haymanot Alem Muche.
